# Understanding supply sustainability of plasma‐derived medicinal products: Drivers and consequences of shortages

**DOI:** 10.1111/vox.70052

**Published:** 2025-05-26

**Authors:** Miriam Belmonte, Anna Albiero, Filip Callewaert, Julien Patris, Amanda Whittal

**Affiliations:** ^1^ Dolon Ltd. London UK; ^2^ argenx Ghent Belgium

**Keywords:** intravenous immunoglobulins, plasma supply, plasma‐derived medicinal products, shortages, sustainability

## Abstract

Plasma‐derived medicinal products (PDMPs), particularly immunoglobulins (Igs), are essential treatments for numerous diseases, often serving as the primary therapeutic option and playing a critical role in patient care. The human origin of these products, however, can lead to supply constraints due to a lack of plasma collection, market dynamics, regulatory challenges and manufacturing complexities. Many nations lack plasma self‐sufficiency and often rely on the United States, which supplies approximately 70% of the world's plasma. This supply chain is vulnerable to disruptions, such as those caused by COVID‐19. Additionally, plasma processing timelines are lengthy—Ig manufacturing takes 7–12 months compared with 2–3 months for biologics. Despite the global Ig market's projected growth from $13.36 billion to $24.98 billion between 2023 and 2032, plasma shortages persist. The European Medicines Agency anticipated shortages to affect 14 European countries in 2024. These factors can have significant implications for patients, with growing demand likely leading to supply challenges and forcing countries to prioritize certain indications in the face of shortages. Policy interventions may be needed to ensure the sustainable use of these products in treating immune‐mediated disorders and related conditions. Exploring alternative treatments where possible could also mitigate the risk of shortages and maintain access to these life‐saving therapies. This review examines the sustainability of PDMPs, focusing on drivers and consequences of shortages, insufficient plasma collection, vulnerability of the plasma supply chain and impacts on patients. A scoping literature research was conducted in PubMed, supplemented by internal knowledge and targeted web searches.

**Highlights**

- The recent increase in demand for plasma, coupled with the intricate process of obtaining plasma‐derived medicinal products (PDMPs) from human sources, regulatory challenges and market dynamics, has resulted in recurring supply shortages.
- Supply constraints of PDMPs, especially intravenous immunoglobulins (IVIgs), can result in prioritization protocols, which may lead to some patients having to discontinue treatment or switch to alternatives.
- Policy interventions and the use of alternative treatments where possible may help better ensure the sustainable and effective use of IVIgs in managing immune‐mediated disorders and related conditions and reduce the risk of shortages.

## INTRODUCTION

Plasma‐derived medicinal products (PDMPs), particularly immunoglobulins (Igs), play a crucial role in treating rare, severe, often genetically inherited conditions such as primary immunodeficiencies (PID), haemophilia, alpha‐1 antitrypsin deficiency (AATD) and hereditary angioedema (HAE) and neurological disorders such as chronic inflammatory demyelinating polyradiculoneuropathy (CIDP), myasthenia gravis (MG), multifocal motor neuropathy (MMN) and Guillain‐Barré syndrome (GBS) [1, 2, 3]. In recent years, the use of these products has increased due to more PDMPs gaining approval for the treatment of additional medical conditions and off‐label use in some cases [4, 5].

Many PDMPs have been identified by the World Health Organization (WHO) as essential medicines [6]; meeting the increasing demand for them is constrained by the limited availability of human plasma [7], which raises concerns about the sustainability of their use and the resulting impact on patient care. In this review, we examine the sustainability of PDMPs and Igs for patients and healthcare systems, including the drivers and consequences of shortages. Information was collected through a scoping literature review in PubMed (cut‐off date: April 2024) and web searches.

## MATERIALS AND METHODS

**FIGURE 1:**
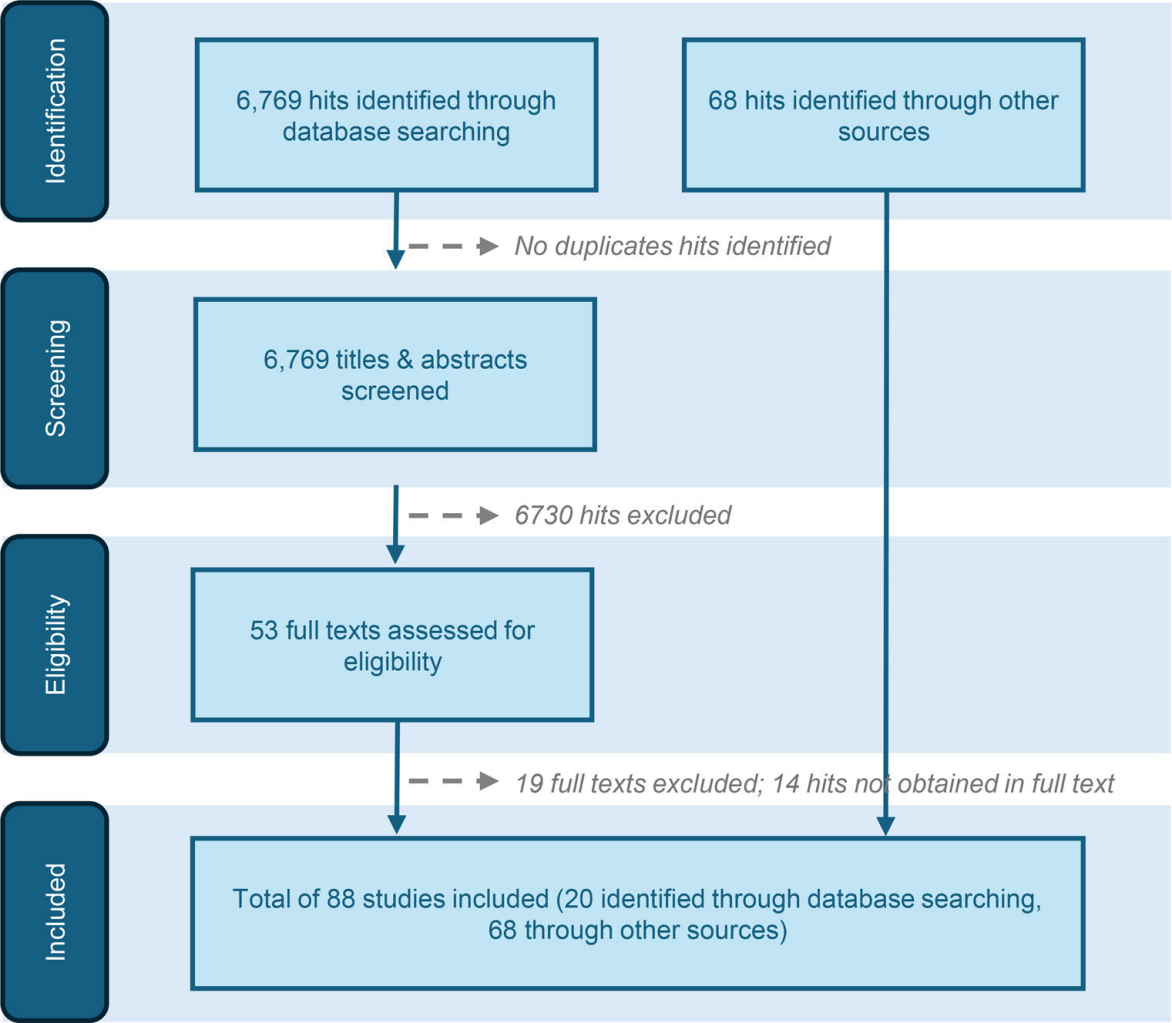
Flow diagram of identifying and selecting studies for inclusion in the paper.

A scoping literature review was conducted to identify publications on the societal and economic impact of PDMPs and IVIgs (cut‐off April 2024). The search, limited to 2008–2024, used the string: ‘(“Plasma‐derived product*” OR “intravenous immunoglobulin*” OR “immunoglobulin*” OR “IVIg*”) AND (“supply” OR “safe*” OR “econom*” OR “burden” OR “shortage*”)’. After removing duplicates, two authors screened the titles and abstracts, excluding studies that focused on clinical aspects of PDMPs/IVIgs, were outside Europe, the United States, Canada, Australia or not in English. From 53 shortlisted citations, 20 were included after full‐text assessment. Additional sources (68) brought the total to 88 articles (Figure 1).

## RESULTS

### Plasma sourcing and PDMP manufacturing

The production of PDMPs begins with the collection of human plasma from healthy donors [3]. Plasma can be obtained through two primary methods: whole blood donations, where plasma is separated from the cellular components (recovered plasma), and aphaeresis, a process that exclusively extracts plasma (source plasma) while returning the cellular components to the donor [3]. The obtained plasma is then ‘fractionated’, that is, separated into individual proteins that can be used for medicinal purposed [3].

To ensure a continuous supply of plasma for fractionation and uninterrupted production, manufacturers rely on a global network of plasma collection and manufacturing [3]. In many countries, including Australia, New Zealand, the United Kingdom, the Netherlands, France, Italy and Spain, plasma collection for fractionation is solely managed by public or non‐profit sectors [3, 8, 9]. Conversely, the United States, Austria, Czechia and Germany allow both public and non‐profit sectors, as well as commercial private plasma collection centres [3] to operate (Table 1).

**TABLE 1:** Method of plasma collection: overview of plasma collection practices by country [3].

Country	Method of plasma collection	
	Public or non‐profit	Private (financial compensation and other incentives provided to donors)
Australia	✓	
Austria	✓	✓
Czech Republic	✓	✓
France	✓	
Germany	✓	✓
Italy	✓	
Netherlands	✓	
New Zealand	✓	
Spain	✓	
United Kingdom	✓	
United States	✓	✓

This table focuses on plasma collection and cannot be interpreted as PDMP self‐sufficiency, as it does not capture elements such as the fact that the plasma collected within a country may be sent for fractionation overseas. Fractionation practices vary widely across countries depending on self‐sufficiency needs and existing fractionation contracts, making them too complex to simplify in a table.

Abbreviation: PDMP, plasma‐derived medicinal product.

The countries that allow both public and non‐profit sectors, as well as commercial private plasma collection centres, achieve higher collection volumes per capita [10, 11]. Due to the complex, multi‐factorial nature of the PDMP supply chain, however, it is important not to draw conclusions on the relative effectiveness of public and non‐profit versus commercial private collection. Moreover, adequate regulations to ensure donor safety are equally important to increasing collection volume.

Fractionation practices differ across countries based on self‐sufficiency goals, healthcare policies and reliance on domestic or international plasma supply. These processes are complex, with some nations like Australia, New Zealand, the United Kingdom, the Netherlands and several others prioritizing domestic fractionation, while others depend on agreements with multinational fractionators for plasma processing [12].

The United States is currently the major global supplier of plasma, providing approximately 70% of the plasma used worldwide [4, 7, 13, 14]. This dominance is due to several factors, including the fact that the United States has the largest share of the world's plasma donation centres (80%), favourable regulations and donor compensation that incentivizes donors to contribute plasma more frequently [15]. In 2020, the Asia‐Pacific region supplied 18% of the global plasma supply, with China providing 75% of this amount. Europe accounted for 14%, while Latin America, the Middle East and Africa combined contributed only 1% [16].

The journey from plasma donation to the administration of PDMP treatments includes multiple complex steps and is extensive, taking between 7 and 12 months, compared with 2–3 months for biologics [3, 7, 17]. This extended timeline means that the PDMPs needed today began production up to a year ago. Moreover, manufacturing and raw materials account for 57% of the total cost for PDMPs, in comparison to 14% for biologics [17]. While subcutaneous administration of Ig is available (SCIg), intravenous administration remains the most commonly used form [18, 19, 20, 21]. In 2017/2018, both Canada and England reported around five times more usage of intravenous immunoglobulins (IVIgs) compared with SCIg (approximately 5 million grams of IVIg vs. 1 million grams of SCIg) [21].

For consistency from this point forward, we will therefore use the term IVIg to broadly refer to Ig.

### Increasing worldwide demand for plasma and PDMPs

The global PDMP market has experienced consistent growth across the globe, expanding at an average annual rate of 7.4% from 1996, when it was valued at $4.8 billion, to $26.6 billion in 2020 [16]. In Europe, the PDMP market share in 2016 was distributed across plasma proteins as follows: IVIg 47.3%, albumin 15.7%, factor VIII 7.6%, hyperimmunes 4.7% and factor IX 2.5%, with all other plasma proteins comprising 23.2% [16]. To meet the anticipated growth in demand, companies have been increasing plasma collection by an average of 8%–9% per year [16].

IVIgs have been the main driver of the global plasma market since the 1990s [16, 22]. In 2016, they represented 47% of the global plasma protein market, following a steady increase from 20% in 1984, 24% in 1996 and 46% in 2008 [23]. The global IVIg market size was estimated at $13.36 billion in 2023 and is projected to reach approximately $24.98 billion by 2032 [24]. The yearly increase in global IVIg usage, as reported by the Marketing Research Bureau, showed an average annual growth of approximately 12% from 2010 to 2018 [5, 25]. Overall, the consumption of IVIgs per 1000 inhabitants rose from 40.4 g in 2010 to 94.6 g in 2021 [26].

**FIGURE 2:**
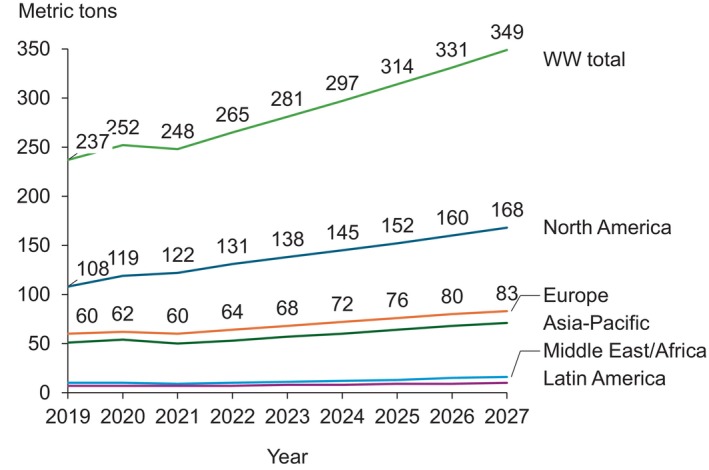
Worldwide (WW) intravenous immunoglobulin demand from 2019 to 2027 (estimated). Figure re‐adapted from Galduf et al. [27].

Research by Galduf et al. shows a steady increase in IVIg demand across regions, based on industry projections. The upward trend is expected to continue over time, with demand in Europe anticipated to rise from 60 metric tons in 2019 to a projected 83 metric tons by 2027 (Figure 2) [27].

The growth in the IVIg market size is largely driven by its application to an expanding number of diseases, including primary and secondary immune deficiencies such as idiopathic thrombocytopaenic purpura, MG and CIDP [28, 29, 30, 31]. Along with the approval of IVIg for the treatment of additional medical conditions, there has been off‐label use [5]. For instance, off‐label use rates in France are approximately 30%, with similar trends observed in other countries [32].

### Plasma supply complexities and IVIg shortages

Many countries outside the United States struggle to be completely self‐sufficient in plasma collection [21]. In the context of this paper, ‘plasma self‐sufficiency’ refers to a country's ability to meet its demand for PDMPs entirely through its domestic plasma collection, without relying on imports. Across Europe, domestically collected plasma meets only about 63% of the demand for PDMPs, with the remainder predominantly imported from the United States [3, 5]. In Europe, 62% of plasma is collected by public collection services, while the private sector, concentrated in Austria, Czechia, Germany and Hungary, collects the remaining 38% [33]. There is significant variation in self‐sufficiency and its definition among individual countries. For instance, France collects 810,000 L of plasma annually and is considered 33% self‐sufficient [21], while Italy, with 880,000 L, is deemed 70% self‐sufficient [34], but still relies on foreign plasma for around 30% of its needs [35]. Spain, collecting 410,000 L, is regarded as 43% self‐sufficient [35].

From 1998 until 2021, the United Kingdom was entirely dependent on imported plasma due to the bovine spongiform encephalopathy (BSE) epidemic [36, 37] and has set a target to reach 30% self‐sufficiency by 2025 [38]. Canada, which is the second highest per capita consumer of IVIg products globally, covers less than 20% of its domestic requirements with its own plasma [39, 40] and spends 66% of the total blood expenditure on supplying these products from outside sources [40]. Australia also has one of the highest per capita uses of IVIg in the world [41] and sources less than half (48%) of its needs domestically [41, 42].

These variations in country self‐sufficiency may arise from different regulatory policies. Countries that have invested more in infrastructure are likely to have higher collection rates. Those that provide compensation for donations (e.g., Germany and Austria) often collect more plasma than those adhering to voluntary, non‐remunerated models (e.g., France and Spain). Additionally, some countries may depend on imports due to historical challenges, as seen in the United Kingdom's past plasma collection ban [37].

IVIgs have been listed as critical medicines by the European Medicines Agency (EMA) for 2023 and 2024 [31]. In recent years, widespread shortages of PDMPs have been reported, with IVIg in particular representing the third top medicinal product affected by shortages across Europe [31, 43, 44]. The EMA has highlighted significant shortages across the European Union (EU) and European Economic Area (EEA), driven by multiple factors, including the recent and consistent rise in demand [31]. These shortages impact IVIgs authorized at both EU/EEA and national levels and were anticipated to persist throughout 2024 [31].

Multiple Organisation for Economic Co‐operation and Development (OECD) countries, including the United Kingdom, France, Greece, Latvia, Lithuania and Portugal, have faced IVIg shortages due to insufficient supply and market withdrawals [45]. Even countries with substantial commercial plasma sources, such as Germany, Czechia, Hungary and the United States, have not been spared IVIg shortages [45, 46]. Documented IVIg shortages worldwide have often resulted in dose reductions, treatment discontinuations or modifications [47] (Table 2).

**TABLE 2:** Documented cases of intravenous immunoglobulin shortages (not exhaustive). Underlying causes and consequences of shortages that occurred between 2017 and 2022.

Year	Documented cases of IVIg shortages (not exhaustive)			
	Country	Disease affected	Causes	Impact
2017	France [48]	CIDP, LSS, MMN, MG	Additional indications for IVIg, production restrictions of the largest pharmaceutical group	Treatment (Tx) delay/discontinuation/modification, dose reduction, clinical deterioration of patient status
2018	Cyprus [49]	PID	NR	Tx dose reduction/discontinuation
	United Kingdom [50]	PID	Demand higher than forecasted, low tender prices/manufacturers allocating IVIg to countries with higher prices, withdrawal of IVIg product Kiovig (Shire)	Tx discontinuation/modification
	Romania [51]	PID	Low tender prices, national pricing policies, newly introduced clawback tax (increased costs of Tx providers)	Increased vulnerability of patients to life‐threatening infections, one patient death
2019	United States [52, 53]	NR	Additional indications for IVIg, off‐label use	NR
2020	Portugal [54, 55]	PID	Plasma collection decreased by ~50% COVID‐19 pandemic, low tender prices	Warning from government agency for human medicines and health products to hospitals to use IVIg only in cases where there is no alternative treatment (e.g., PID)
	Spain [16]	NR	NR	Tx delay/dose reduction/modification
2021	France [56]	PID, SID	NR	Exceptional and temporary importation of products that are not normally in the French market
2022	Poland [57, 58, 59]	NR	COVID‐19 pandemic, low tender prices, lack of national plasma fractionation	Tx dose reduction/discontinuation/modification
	Romania [60]	PID, neuro‐conditions	Reduced supply of products on the market	78% of interviewed doctors had to reduce Tx dose by >30%; PID: >2–3 months Tx discontinuation (17%–21%), Tx delay; neuro‐conditions: >2 months Tx discontinuation (16%), >3 months Tx discontinuation (71%)
	Latvia [61]	NR	NR	NR

Abbreviations: CIDP, chronic inflammatory demyelinating polyradiculoneuropathy; IVIg, intravenous immunoglobulin; LSS, lumbar spinal stenosis; MG, myasthenia gravis; MMN, multifocal motor neuropathy; NR, Not reported; PID, primary immunodeficiencies; SID, secondary immunodeficiencies.

Altogether, these data show that the heavy reliance on external plasma presents a potential challenge to the global plasma supply chain and highlights the pressing need for strategies to enhance supply resilience and meet growing demand.

### Additional factors exacerbating vulnerability in plasma sourcing

In addition to the demand and supply imbalances, other factors have exacerbated the shortages of PDMPs.

A key issue is the vulnerability of the supply chain to external disruptions. Unexpected events, such as the COVID‐19 pandemic, have significantly impacted plasma collection and contributed to shortages [47]. While many medicines faced supply chain disruptions during the COVID‐19 pandemic [62], several factors exacerbated the impact on IVIg in particular. Social restrictions, health concerns and decreased donor willingness [13, 63] caused a significant decline in blood product donations, further worsened by fears of virus exposure, actual COVID‐19 infections among potential donors and reduced collection centre capacities due to safety measures [12, 64]. Moreover, plasma intended for IVIg production was often redirected to clinical trials or research [13, 63], adding another layer of demand that further strained an already pressured system.

The unique dynamics of the PDMP supply chain further exacerbate its vulnerability to external disruptions. Unlike many other medications, the supply of IVIg is heavily reliant on plasma collected from the United States [3, 5], making it particularly susceptible to shortages when the supply chain is disrupted. In the United States, the pandemic caused a 20% reduction in plasma donations, from 53,532,216 in 2019 to 43,800,749 in 2021, leading to a shortage of raw materials for PDMPs [4, 65]. Similarly, most EU countries experienced a median decrease of about 9% in blood and blood component donations during the early months of the pandemic [63]. For example, France saw an 11% reduction in IVIg production and supply to hospitals by December 2021, and the overall plasma self‐sufficiency rate in Europe fell to 15% in 2022 [16]. The global plasma supply's strong reliance on the United States can create additional risks since restrictions to trade due to economic, political or public health reasons may have an impact on global access to PDMPs. Even regional events, such as the recent closure of border crossings with Mexico, can impact plasma collection and the global supply chain [66].

Tendering practices also impact the availability of therapies, including PDMPs. Many countries establish tendering agreements with IVIg manufacturers to secure specific volumes of supply of Igs. Since IVIgs are derived from plasma, tender specifications determine the supply chain's capacity to meet demand [19]. Inadequate tendering practices, such as setting prices too low or inaccurately forecasting demand, can lead to therapy shortages. This has been observed in countries like the United Kingdom [19, 50] and Romania [19, 51], where suboptimal tendering practices have disrupted the supply chain and contributed to therapy shortages.

Altogether, these factors create a complex web of challenges that exacerbate the vulnerability of plasma sourcing, underscoring the urgent need for a more resilient and equitable global strategy to ensure consistent access to these essential treatments.

### Impact of shortages on patients' access

National authorities have implemented measures to mitigate the impact of shortages. For instance, several national agencies have issued guidelines to ensure IVIg is used primarily for specific conditions, thereby reducing the risk of patients being denied access due to off‐label use [25, 29, 67]. The French National Agency for Medicines and Health Products Safety (ANSM) recently alerted healthcare professionals to adhere to these prioritization guidelines [56, 68]. Additionally, patient concerns have been raised about the potential consequences of these measures, with the Association against Peripheral Neuropathies patients' association urging vigilance regarding the risks posed by the widespread use of certain alternatives to IVIgs [69]. In the United Kingdom, a 38% plasma deficit in 2020 led the National Health Service (NHS) to introduce measures to allocate IVIg to patients with the highest clinical need [38]. Similarly, various EU countries have developed national guidelines to prioritize treatment for serious pathological conditions during shortages [70, 71, 72, 73].

While this approach can help to mitigate the impact of limited supplies, it also means some patients may face delays or reduced access to IVIg [64, 70, 71, 72, 73]. Moreover, it can lead to modifications of patients' treatment regimens, which can result in delays, decreased doses or the need to switch to alternative and, in some cases, suboptimal treatments [64, 70, 71, 72, 73].

When IVIg becomes unavailable or access is restricted, patients may have to transition to different IVIg formulations or alternative treatments, which can negatively impact their health [48]. A study conducted in France at the University Hospital, la Timone, Marseille, found that out of 142 patients with CIDP, MG or MMN, 111 (78%) experienced modifications to their IVIg treatment [48]. Of the 99 patients analysed, 58 showed clinical score deterioration, with 31 experiencing moderate to clinically significant declines in their condition [48]. Best practice guidelines recommend avoiding such switches unless clinically necessary to prevent adverse reactions and minimize the risk of batch‐associated infections [74]. Common side effects from switching IVIg products include headaches, chills and fatigue [75]. In the United Kingdom, changes in commissioning guidance and supply issues in 2017–2018 led to numerous patients switching between products, resulting in both local and systemic adverse reactions [74].

These challenges are not isolated; they reflect a broader issue revealed by an international survey conducted across the United States, Canada, Europe, Japan and Australia [43]. The survey highlighted a significant lack of well‐developed mitigation strategies and clinical guidance to effectively manage IVIg shortages. These challenges underscore the necessity for improved measures to prevent and manage IVIg shortages, safeguarding patient health and maintaining treatment efficacy during periods of limited supply.

### Regulatory reforms and their impact on plasma supply

The need for improvements in preventing and managing IVIg shortages has been recognized. In April 2024, the European Commission approved a new regulation under the Substances of Human Origin (SoHO) framework to enhance the quality and safety standards for human‐origin substances, including plasma [76, 77]. Definitively adopted in August 2024 [78], this regulation aims to ensure a more reliable and sustainable plasma supply across Europe by encouraging member states to develop effective plasmaphaeresis programmes and expand plasma collection and fractionation capabilities [79, 80]. It also seeks to foster collaboration among public, private and non‐profit sectors to boost plasma collection efforts [79, 80].

However, the impact of the regulation on IVIg availability remains uncertain. Under the new regulation, the principle of ‘financial neutrality’ should be applied to ensure donation does not result in financial gain for the donor or constitute an incentive to donate [78, 81]. Such restrictions could affect recruitment in countries where financial incentives have previously increased donation rates [19]: Austria, Czechia, Germany and Hungary, where financial compensation for plasma donation is allowed, collectively contribute over 55% of the plasma collected in Europe for manufacturing PDMPs (whereas countries, like Italy, France and Spain, where donor compensation is not allowed, primarily use their collections domestically) [3]. The new regulation might therefore hinder donor recruitment in these regions, impacting overall plasma availability. At the same time, the regulation plays a critical role in protecting donor health [78]. Overly frequent donations can be an issue in countries that offer compensation, which can negatively impact donor health, as well as patient health if donors fail to disclose health risk factors [78]. There is a delicate balance to be struck between incentivizing donations to protect against shortages and protecting donor health. It is thus important to look for other ways to manage plasma shortage risks beyond increasing donations.

Moreover, the European Commission has acknowledged that the new legislation does not adequately address the ongoing challenges in plasma supply, leaving patients vulnerable to shortages [63, 82]. The implementation of EU‐level contingency plans for blood and plasma is crucial, yet there is scepticism about their effectiveness in mitigating supply interruptions, especially given Europe's reliance on plasma imports from the United States [76]. While the regulatory reforms aim to enhance plasma supply and safety, these remaining challenges around supply stability underscore that there is still a need for additional strategies to improve plasma sustainability and reduce Europe's reliance on external sources.

### Discussion and summary

PDMPs, including IVIgs, are crucial therapies for numerous conditions [30], but the accessibility and sustainability of their production and supply are variable. This review explored the complexities of plasma supply, the increasing demand for PDMPs [5, 23, 25], particularly IVIgs and the factors exacerbating vulnerabilities in plasma supply chains that can impact patients globally.

A key limitation of this review is its focus on developed regions with established PDMP systems, excluding regions like Latin America, the Middle East and Africa, which contribute only 1% of the global plasma supply [16]. The scarcity of published data on PDMPs from regions with low plasma contribution limited the scope of this analysis and highlights a need for further research dedicated to these regions.

A study conducted in Africa identified several organizational, financial and socio‐economic barriers to blood availability, including a high prevalence of transfusion‐transmitted infections due to inadequate donor screening, lack of privacy during donation, a high proportion of one‐time donors, complex epidemiological challenges, inadequate government funding, a shortage of trained personnel, misconceptions about blood donation risks and spiritual or religious beliefs [77]. Similar inadequate infrastructure, regulatory barriers and financial constraints may be contributing to the low plasma supply in other low‐income regions.

Addressing these challenges requires a multifaceted approach, including international collaboration, capacity‐building initiatives and policy reforms tailored to regional needs. Strengthening these efforts could help ensure more equitable access to PDMPs worldwide.

Within the countries in scope for this review, the key factors that impact PDMP sustainability and accessibility include

1. Rising demand from expanding disease indications: The broadening clinical applications for PDMPs are driving a significant rise in demand for IVIg [5, 29, 30]
2. Vulnerability to shortages: the extensive timeline required to produce PDMPs [3, 17] necessitates a steady and substantial flow of plasma donations. This process is vulnerable to fluctuations in donation rates and external factors such as regulatory changes or global events, potentially leading to shortages if demand surges unexpectedly or supply chains are disrupted [31, 43, 44, 45, 46, 47]. Additionally, a heavy reliance on imported plasma, primarily from the United States, accentuates the global supply chain's vulnerability to shortages [4, 13, 14].

IVIg shortages, stemming from the intrinsic nature of its production and structural supply–demand factors, can result in treatment delays, modifications or shifts to alternative therapies [47, 64, 70, 71, 72, 73]. The reliance on consistent plasma availability highlights the need for strategies to enhance supply resilience and meet growing clinical demands. Policymakers are striving to improve PDMP regulation, but the effectiveness of these measures remains uncertain. In Europe, the recently implemented SoHO legislation aims to improve plasma supply standards and ensure adherence to ethical principles that protect human dignity and promote altruistic donations [78, 81]. At the same time, it has potential limitations in addressing the core issues related to plasma sourcing and PDMP production. In particular, a lack of ‘fair’ financial incentive to donate plasma could reduce the amount of plasma collected in Europe, as the highest plasma collection rates are often seen in countries that provide financial compensation to donors [10, 11]. In addition, the data presented in this review show a growing demand for PDMPs [27] in future years, which will likely keep outpacing the plasma supply required to produce them. Further policies and strategies may therefore be needed to better ensure PDMP sustainability.

Besides policy reforms, alternative and innovative therapies may help alleviate some of the pressure of PDMP shortages. While the critical role of IVIgs for many conditions will remain, innovations in drug therapies for some conditions may offer promising avenues to reduce reliance on blood‐derived products. For example, haemophilia, a genetic bleeding disorder, traditionally required regular infusions of clotting factor concentrates from donated plasma [83]. Recent advancements in drug therapies, including recombinant and extended half‐life factors, have significantly reduced the need for plasma‐derived products, thus decreasing reliance on human blood donations [83]. Similarly, the use of hypomethylating agents (azacitidine, decitabine) [84], lenalidomide [85] and luspatercept [86] have reduced the reliance on frequent blood transfusions in myelodysplastic syndrome (MDS) patients, while gene therapies are also gradually developing [87]. More recently, neonatal Fc receptor‐targeted therapies have been approved and are being used in clinical practice in neurology and haematology indications [88] and hold promise for broader application in various antibody‐mediated autoimmune diseases [89, 90]. These therapies specifically target the Fc receptor, inhibiting the degradation of immunoglobulin G (IgG) and albumin, which prolongs their half‐life in the bloodstream [88, 91]. As a result, they could offer an alternative to high‐dose IVIg, thereby helping to alleviate treatment burden on both healthcare systems and patients [88]. Altogether, these examples underscore the potential of medical advancements as one potential part of the solution to alleviate pressure on the plasma supply chain and enhance treatment options for patients, contributing to more sustainable healthcare solutions.

Ensuring the sustainability and accessibility of essential PDMPs, like IVIg, requires addressing challenges in plasma sourcing and distribution. Supporting plasma collection, streamlining production, ensuring fair pricing and implementing effective policies are essential. Moreover, introducing alternative and innovative therapies where medically justified can reduce reliance on blood‐derived products and ease pressure on the plasma supply chain. These efforts can help ensure better sustainability of PDMPs, allowing patients to count on consistent access and continuity of care.

## CONFLICT OF INTEREST STATEMENT

J.P. and F.C. are employees of argenx. M.B., A.A. and A.W. have received consultancy fees from argenx. argenx is a global immunology company committed to improving the lives of people suffering from severe autoimmune diseases; Dolon is a strategic pricing and market access consultancy specializing in rare and severe diseases.

## Data Availability

Data sharing is not applicable to this article as no new data were created or analysed in this study.
